# The Effect of Protozoa Indigenous to Lakewater and Wastewater on Decay of Fecal Indicator Bacteria and Coliphage

**DOI:** 10.3390/pathogens12030378

**Published:** 2023-02-25

**Authors:** Asja Korajkic, Brian R. McMinn, Valerie J. Harwood

**Affiliations:** 1 United States Environmental Protection Agency, 26W Martin Luther King Jr. Drive, Cincinnati, OH 45268, USA; 2 Department of Integrative Biology, University of South Florida, 4202 E Fowler Avenue, Tampa, FL 33620, USA

**Keywords:** predation, fecal indicator bacteria, bacteriophage, ambient water, wastewater

## Abstract

Fecal indicator bacteria (FIB: *Escherichia coli* and enterococci) are used to assess recreational water quality. Viral indicators (i.e., somatic and F+ coliphage), could improve the prediction of viral pathogens in recreational waters, however, the impact of environmental factors, including the effect of predatory protozoa source, on their survival in water is poorly understood. We investigated the effect of lakewater or wastewater protozoa, on the decay (decreasing concentrations over time) of culturable FIB and coliphages under sunlight and shaded conditions. FIB decay was generally greater than the coliphages and was more rapid when indicators were exposed to lake vs. wastewater protozoa. F+ coliphage decay was the least affected by experimental variables. Somatic coliphage decayed fastest in the presence of wastewater protozoa and sunlight, though their decay under shaded conditions was-10-fold less than F+ after 14 days. The protozoa source consistently contributed significantly to the decay of FIB, and somatic, though not the F+ coliphage. Sunlight generally accelerated decay, and shade reduced somatic coliphage decay to the lowest level among all the indicators. Differential responses of FIB, somatic, and F+ coliphages to environmental factors support the need for studies that address the relationship between the decay of coliphages and viral pathogens under environmentally relevant conditions.

## 1. Introduction

Enteric viruses have been identified as the main etiological agents of waterborne illness in recreational settings [1,2,3,4]. However, the sanitary quality of recreational waters is routinely and most commonly assessed by enumeration of culturable fecal indicator bacteria (FIB), such as *Escherichia coli* and enterococci, and more recently quantitative polymerase chain reaction (qPCR) in the United States [5]. Despite a long history of use as an indicator of fecal contamination, there are many criticisms of the FIB approach, at least partially due to the different fate and transport characteristics of FIB and viral pathogens (recently reviewed in [6,7,8]). For example, the decay of FIB in ambient waters is generally faster than that of viral pathogens due to greater susceptibility to a range of biotic and abiotic environmental factors. If indicators of fecal contamination are to accurately predict human health risk, the decay rate of indicators and pathogens in aquatic environments should be similar, or at least predictably related [8].

In recent years, there has been renewed interest in bacteriophages, such as *E. coli-* infecting coliphages, for many different applications [9,10,11,12], including as viral indicators of fecal pollution in recreational waters [13] due to their many similarities to enteric viral pathogens [14]. Coliphages infect *E. coli*, a commensal human gastrointestinal tract species, and are subsequently shed in feces by hosts, following routes of dissemination into the environment that are similar to those of enteric viral pathogens. The utilization of coliphages in this context is further supported by epidemiological studies demonstrating the association between coliphage levels and gastrointestinal illness in recreational bathers [15,16,17,18]. However, additional information regarding the effect of various biotic and abiotic environmental factors on FIB and coliphage decay [19,20,21,22] in aquatic habitats is needed to assist with future recreational water quality criteria (RWQC) development. While the effect of some parameters (e.g., temperature) is well characterized, others (e.g., microbial interactions including viral lysis, intra- and interspecies competition, and protozoan predation) are less well understood [8].

Protozoan grazers, which consume bacteria and viruses, are a vital part of microbial food webs in aquatic habitats [23], and these top-down processes are especially important in oligotrophic environments [24,25,26], such as William H. Harsha Lake, the subject of this study [27,28]. The effect of protozoan grazing on FIB has been documented in several studies, mainly utilizing singular, laboratory-cultured strains of FIB [29,30,31,32,33]. A limited number of studies relied on more realistic sources of FIB (e.g., human and animal feces and wastewater), however, FIB sources in these studies were not manipulated and therefore contained protozoan predators that are ubiquitous in such environments (e.g., *Blastocystis, Entamoeba, Bodo, Colpidium* spp.) [34,35,36]. These studies consistently found a greater decay of FIB in the presence of aquatic protozoa populations, however, the magnitude of the predator effect was reduced compared to studies that used FIB cultured in the laboratory [20,21,22,37]. The presence of protozoan communities indigenous to feces and/or wastewater in the inoculum may have contributed to the difference in magnitude of the protozoan effect on FIB decay in these experiments that was not explored in the studies.

The role of protozoan predation on the decay of viruses, including coliphage, is not as clear and has been studied less frequently [8]. However, some controlled laboratory feeding studies indicated the ingestion of T4 and MS2 coliphages by *Tetrahymena*, *Thaumatomonas*, and *Salpingoeca* spp. [38,39]. Earlier studies generally indicated faster decay of enterophage (bacteriophage infecting enterococci), F+, and somatic coliphage in the presence of autochthonous aquatic protozoan communities compared to autoclaved and filtered river, lake, and marine waters [19,40,41]. However, similar to some FIB studies, these observations were recorded for either singular bacteriophage strains or bacteriophage cultivated from wastewater, thereby eliminating any possible effect of predatory protozoa from wastewater or feces. The only field study utilizing wastewater and human feces as a source of bacteriophages (somatic and F+ coliphages, GB-124 bacteriophage infecting *Bacteroides fragilis*) noted minimal effects of marine protozoan communities on decay as compared to filtered marine water controls [20]. Furthermore, this was the only study that contained wastewater protozoan communities contributed by the inoculum, making it unclear whether the effect of protozoa autochthonous to ambient waters observed in earlier studies was confounded by the presence of wastewater protists, or whether the source of the bacteriophage (i.e., laboratory propagated strains vs. wastewater/feces) influenced the results.

Unlike the effect of the source of predators, the contribution of ambient sunlight to the decay of FIB and coliphages has been more extensively documented and is reviewed in [8]. In general, culturable FIB and infectious coliphage exhibit greater decay when exposed to ambient sunlight, compared to dark or shaded controls [8], and this effect is attributed to either direct damage to nucleic acids in the form of pyrimidine dimers caused by UVB radiation or endogenous and/or exogenous photo-oxidative damage caused by UVA radiation [42] although the precise mechanism of UV-induced damage is likely to differ among different species and taxonomic groups.

The slower decay of FIB and coliphages in the absence of any protozoan predators and in dark or shaded conditions compared to sunlight exposure has been documented extensively and reviewed in [8,42,43,44,45]. However, the effect of protozoa source has not been studied before. Therefore, we opted to focus on the effect of protozoan predators from lakewater vs. wastewater and potential interactions with ambient sunlight instead. Through selective removal of protozoan communities from either the lakewater or the wastewater, we were able to investigate the effects of different grazer sources on the decay of the diverse communities of FIB and coliphage contained in lakewater and wastewater. Incubation under sunlight and shaded conditions enabled characterization of the relative influence of and interactions between predator source and ambient sunlight. Finally, a direct comparison of FIB and coliphage decay characteristics allowed us to document differential and temporal responses of these two fecal indicator groups to biotic and abiotic environmental stressors.

## 2. Materials and Methods

### 2.1. Experimental Design

The goal of the study was to compare the effect of protozoa from lakewater (treatments: A and B) vs. wastewater (treatments: C and D) on decay (log_10_ reduction) of bacterial and viral indicators under conditions of high (treatments A and C) or low (treatments: B and D) light intensity (Table 1). The experimental treatments were as follows: (A) exposure to lake protozoan predators and ambient sunlight (lake protozoa/sun), (B) exposure to lake protozoan predators only (lake protozoa/shade), (C) exposure to wastewater protozoan predators and ambient sunlight (wastewater protozoa/sun) and (D) exposure to wastewater protozoan predators only (wastewater protozoa/shade) (Table 1). A submersible aquatic mesocosm (SAM) device, constructed as previously described [8,19,22,46,47] was used to conduct the experiment in situ. Even though a singular SAM device was deployed for this experiment, each treatment and time point consisted of three independent dialysis bag replicates, prepared as described below, as is common practice for similar field studies, e.g., [20,46,48,49,50]. Fifty percent of bags with each inoculum type (i.e., lake protozoa or wastewater protozoa) were placed at the upper level of the SAM for the sunlight-exposed treatment (approximately 2–5 cm below the water surface), while the remaining half was placed at the lower level (approximately 25-30 cm below the water surface) underneath the heavy-duty black plastic tarp covering to simulate shaded conditions. Independent triplicate dialysis bags for each treatment were collected for enumeration of FIB and coliphages (as described below) immediately after the inoculum preparation (T_0_) and after 24h (T_1_), 72h (T_3_), 120h (T_5_), 192h (T_8_), and 336h (T_14_) of in situ incubation.

Hourly light intensity (lux) and temperature (°C) readings were recorded at both the upper and lower levels of the SAM using HOBO^®^ UA 002-08 data loggers (Onset Computer Corporation, Bourne, MA USA). The mean and standard deviation for the water temperature readings were 16.7 ± 1.2 °C and 16.5 ± 0.90 °C for upper (sunlight) and lower (shade) levels of SAM, respectively. The mean and standard deviation for light intensity measurements were 585.0 ± 3490.0 lux for the upper and 111.2 ± 429.0 lux for the lower level of the SAM. Mean light intensity was significantly higher at the upper level of the SAM compared to the lower level (*p* < 0.0001) indicating that our experimental design adequately created shaded conditions.

### 2.2. Mesocosm Preparation

Primary wastewater effluent and ambient water samples (~15 L each) were collected from a local wastewater treatment plant (Little Miami Wastewater Treatment Plant, Cincinnati, OH: 39.1038889° N, -84.4330556° W) and William H. Harsha Lake (Batavia, OH: 39.0252°N, -84.1303° W), respectively. Immediately after collection, ½ of each sample type was passed through a (0.80 µm pore size, 47 mm diameter) nitrocellulose membrane filter (Pall Corporation, Port Washington, NY USA) to remove protozoan predators and other particulate matter. Removal of protozoa via filtration is a common procedure and less detrimental to the integrity of the water sample compared to other techniques (e.g., heat and chemical treatments) [51,52,53,54,55]. Both filtered and unfiltered samples were held at 4 °C overnight to minimize any changes in microbial populations.

The following day (<24 h after sample collection) the mesocosm inoculum was prepared by mixing a 1:1 ratio of either unfiltered lakewater with filtered primary wastewater effluent (treatments A and B) or filtered lakewater with unfiltered primary wastewater effluent (treatments C and D) (Table 1). Given that we used wastewater as the source of FIB and coliphage, and therefore could not modify the starting concentrations, and accounting for a ~2 log_10_ difference in concentrations between the two indicator types, this particular ratio was chosen to ensure that quantifiable densities could be obtained for a maximum number of sampling time points. Two hundred milliliters of each inoculum type were used to fill regenerated cellulose dialysis bags (75 mm flat width, 13–14 kD pore size MWCO, Spectrum Labs, Rancho Dominguez, CA USA) that were rehydrated for 24 h in sterile diH_2_O prior to the start of the experiment. Potential attenuation of ambient sunlight by the regenerated cellulose dialysis bag material has been tested previously and found to be minimal (<10%) [21]. Prepared dialysis bags were placed in Ziplock™ bags containing approximately 50 mL of ambient water to prevent desiccation and transported to the field site (William H. Harsha Lake) on ice.

### 2.3. FIB and Bacteriophage Enumeration

The FIB and coliphage concentrations were measured using the standard membrane filtration technique [56,57] and double agar layer (DAL) assays [58], respectively. When necessary, decimal dilution series were prepared using a sterile 1X phosphate-buffered saline (PBS) solution (0.0425 g L^−1^ KH_2_PO_4_ and 0.4055 g L^−1^ of MgCl_2_: pH 7.2 Sigma Aldrich, St. Louis, MO). For FIB enumeration, samples were filtered through 0.45 µm (47 mm diameter) nitrocellulose filters and incubated on either mEI for 16–18 h at 41 °C (enterococci) or modified mTEC agar for 2 h at 35 °C, followed by 14–16 h at 44.5 °C (*E. coli*). For the somatic and F+ coliphages, 1 mL of sample was added to 5 mL of the molten top (0.7% agar) tryptic soy agar (TSA) overlay containing 0.1% of appropriate antibiotic stock solution (100 µg mL^−1^ nalidixic acid for somatic or 15 µg mL^−1^ streptomycin/ampicillin for F+ coliphage [Fisher Scientific, Waltham, MA]) followed by the addition of 200 µL of appropriate *E. coli* host (CN-13 ATCC#700609 [somatic] or F_amp_ ATCC#700891 [F+], American Type Culture Collection, Manassas, VA USA) in the midlog growth phase. The top agar overlay mixture was poured on the bottom agar TSA plates (1.5% agar and containing 0.1% of appropriate antibiotic stock solution) and then incubated at 37 °C for 16–18 h. The following day, characteristic colony-forming units (CFU) and plaque-forming units (PFU) were enumerated. During each sampling event, for both FIB and coliphages, method blank (sample substituted with 1X PBS) and media sterility negative controls were performed. For the duration of the study, no CFUs or PFUs were observed indicating the absence of contamination.

### 2.4. Data Analyses

FIB and coliphage concentrations were log_10_ transformed prior to data analyses. The decay of FIB and coliphages was calculated as cumulative log_10_ reduction (log_10_ C_0_–log_10_ C_T_) where C_T_ represents the concentration at different sampling time points (T_1_, T_3_, T_5_, T_8_, and T_14_) and C_0_ represents the starting concentrations measured at T_0_. To facilitate comparisons with other studies, daily decay rates for each organism and treatment are also provided in Table S1. Out of 72 samples collected during the study, the proportion of samples containing no detectable FIB or coliphage was low (i.e., 12.5% for enterococci, 0% for *E. coli*, 11.1% for F+ coliphage, 1.38% for somatic coliphage) and it occurred in the later stages of the experiment (T_5_–T_14_). Table S2 contains details on observations below the detection limits. GraphPad Prism version 8.1.2 (GraphPad Software, La Jolla, CA USA) was used to conduct a two-way analysis of variance (ANOVA) with Tukey’s multiple comparison test to evaluate the effects of the two factors (source of protozoan predators and exposure to ambient sunlight) on decay (Table 2). The same software was used to conduct the one-way ANOVA to compare decay across different indicators within the same treatment and Wilcoxon matched pairs signed rank test to assess differences in light temperature readings between the upper and lower levels of the SAM. The pairing was effective as indicated by a high Spearman correlation coefficient (r = 0.9252) and a low corresponding *p* value (<0.0001).

## 3. Results

### 3.1. Effect of Predator Source and Sunlight on FIB and Coliphage Decay

**Figure 1:**
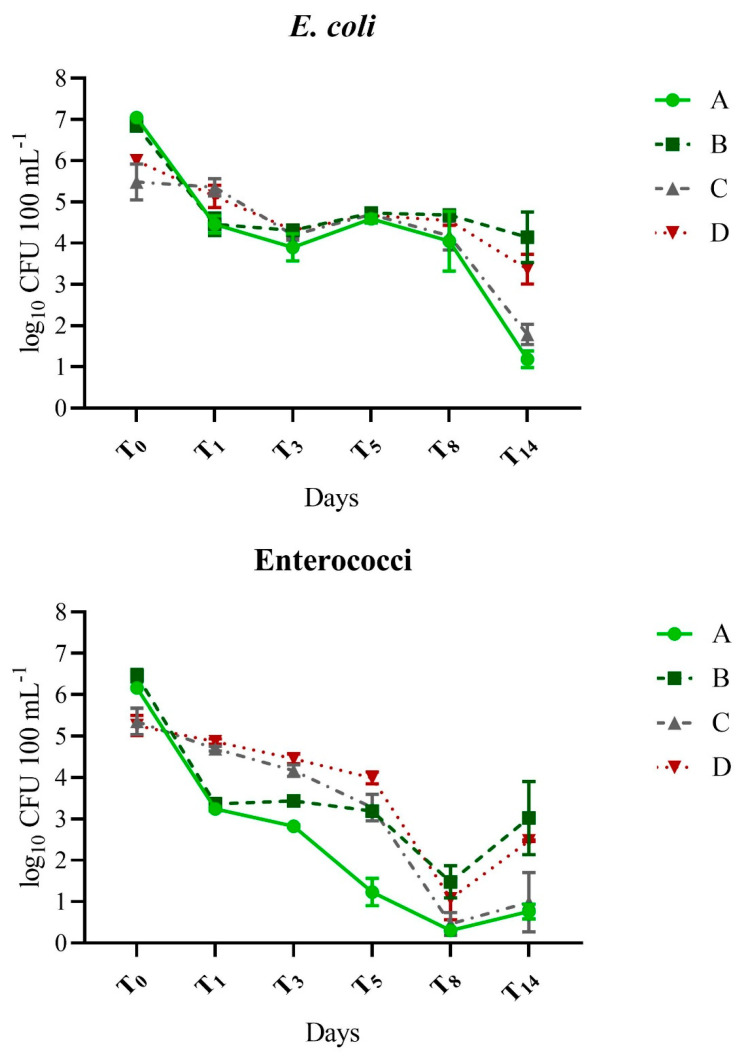
Change in concentrations of *E. coli* and enterococci over time in response to the source of protozoan predators and exposure to ambient sunlight. Treatments: A (lake protozoa/sun), B (lake protozoa/shade), C (wastewater protozoa/sun), D (wastewater protozoa/shade). Error bars represent the standard deviation between independent dialysis bag replicates. All data shown including samples where LOD was used.

The source of protozoan predators had a profound and significant effect on the decay of *E. coli* and enterococci in the first 24 h, from T_0_ to T_1_ (Figure 1, Table 2 and Table 3), accounting for >86% of the observed variability in log_10_ reduction (Table 2). FIB exposed to lake protozoa (A and B) decreased by 2.39–3.10 log_10_ by T_1_, while those exposed to wastewater protozoa (C and D) decreased only 0.13–0.87 log_10_ in the first 24 h of the experiment (Table 3). The influence of protozoan source remained high at T_8_, accounting for 64.7% and 42.6% of variability for *E. coli* and enterococci, respectively (Table 2). At T_14_, the protozoa source was a significant factor in *E. coli* decay, accounting for 20.5% of variability, although it was not significant in enterococci decay. As the influence of protozoan source on decay decreased over time, sunlight became a significant factor, accounting for 57.1% of the variability in *E. coli* decay at T_14_. The interaction of variables was also a significant factor at T_14_, contributing 18.2% of the variability and indicating that the influence of sunlight was dependent on the protozoa source (Table 2). Sunlight became a significant factor in the decay of the enterococci at T_5_ and it remained so until T_14_ when it accounted for 59.7% of the variability (Table 2).

**Figure 2:**
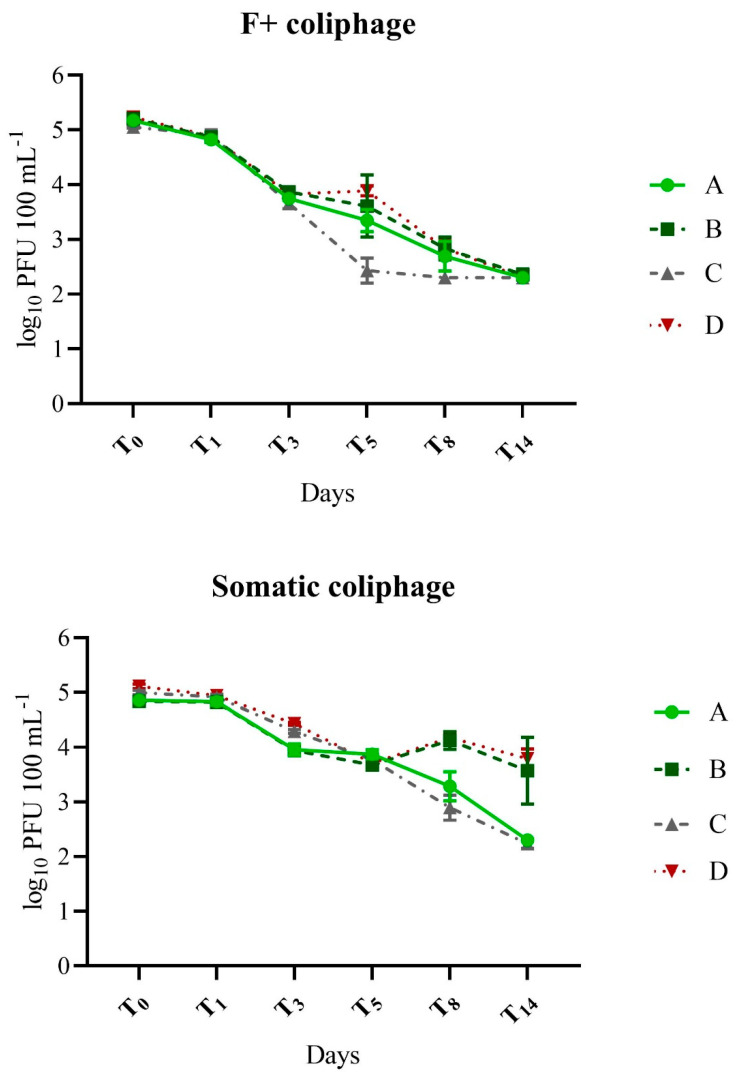
Change in concentrations of somatic and F+ coliphage over time in response to source of protozoan predators and exposure to ambient sunlight. Treatments: A (lake protozoa/sun), B (lake protozoa/shade), C (wastewater protozoa/sun), D (wastewater protozoa/shade). Error bars represent standard deviation-independent dialysis bag replicates. All data shown including samples where LOD was used.

The effect of the source of protozoan predators on the coliphages varied between somatic and F+ coliphages (Figure 2, Table 2 and Table 3). Predator source was not a significant factor in the log_10_ reduction of F+ coliphage, at any of the time points (Table 2). In contrast, the predator source significantly influenced somatic coliphage decay at T1–T8, accounting for the maximum variability of 86.6% at T_3_, though diminishing to 10.7% at T_8_ and becoming a negligible factor at T_14_ (Table 2). Somatic coliphage decay was generally greater in the presence of wastewater protozoa compared to lake protozoa (T_1_, T_5_, and T_8_) except at T_3_ (Table 3), although the magnitude of the difference was not as pronounced as it was for FIB.

The effect of sunlight on bacteriophage decay was different for F+ vs. somatic coliphage, particularly toward the end of the experiment (T_8_ and T_14_). While sunlight was not a significant factor in the decay of either coliphage group until T_5_. At T_8_, sunlight contributed nearly 80% to variability in somatic coliphage decay, though less than half that to F+ coliphage decay (i.e., ~34%) (Table 3). The difference was even more apparent at T_14_ when the effect of sunlight on somatic coliphage increased further to 83%, and log_10_ reduction values in the sun were double the log_10_ reduction values in the shade (Figure 2, Table 3). Sunlight was not a significant contributor to F+ coliphage decay at T_14_, further highlighting disparities in the sunlight effect on the two coliphage groups. In fact, the magnitude difference in the decay of the F+ coliphage in the sun vs. the shade at T_8_ and T_14_ was only a small fraction of that observed for the somatic coliphage. (log_10_ reduction: 0.06-0.22) (Figure 2, Table 3). For example, at T_8_, when sunlight was a significant factor in decay for somatic and F+ coliphages, decay of the somatic coliphage in sunlight was twice that of the shaded conditions (difference of 0.86 and 1.16 log_10_ between sun and shade in the presence of lake and wastewater protozoa, respectively) (Figure 2, Table 3). In contrast, F+ coliphage decay in sun vs. shade at T_8_ differed by only 0.11 and 0.36 log_10_ in the presence of lake and wastewater protozoa, respectively.

### 3.2. Decay of FIB Compared to Coliphage

*E. coli* and enterococci typically decayed more rapidly than coliphages throughout the study (Figure 1 and Figure 2, Table 3,) and this trend was particularly evident in treatments containing lake protozoa (A: lake protozoa/sun and B: lake protozoa/shade). For example, at the last time point, T_14_, log_10_ reduction of FIB in treatment A (lake protozoa/sun), was nearly double that of the coliphages (≥5.4 vs. 2.9). This difference was statistically significant (*p* ≤ 0.0001) when either FIB group was compared to either coliphage group. In treatment B (lake protozoa/shade) at T_14_, enterococci decayed faster compared to somatic coliphage (*p* = 0.0121), though there were no other statistically significant comparisons. While there was a trend for the faster decay of FIB compared to coliphages for treatment C (wastewater protozoa/sun) (Table 2), there were no significant differences in decay among all microorganisms (*p* ≥ 0.1659) at the last time point. Finally, all microorganisms decayed significantly faster (*p* ≤ 0.0126) than the somatic coliphage at T_14_ in treatment D (wastewater protozoa/shade).

## 4. Discussion

Measurement of viable and culturable FIB used historically to assess water quality may not be sufficient indicators of the sanitary quality of recreational waters, considering that the majority of recreational water disease outbreaks are caused by viral pathogens [1,2,59]. Therefore, viral indicators, specifically somatic and F+ coliphage, have been suggested as additional monitoring tools for recreational waters [13]. In order to improve our understanding of the utility of both FIB and coliphage as fecal indicators, more information is needed about their fate in ambient waters.

While the direct effect of protozoan grazing on the decay of FIB and coliphage has been well documented [8,23,44], the effect of protozoa source on indicator decay remains unexplored. To the best of our knowledge, this is the first field study where the effect of predator source on the decay of fecal microorganisms was investigated through systematic removal of protists from either the wastewater inoculum or lakewater medium.

The source of predators had a significant impact on the decay rates of *E. coli* and enterococci, as FIB decay in the presence of lake predators was frequently ≥1 log greater than in the presence of wastewater protozoa. The protozoan communities autochthonous to lakewater and wastewater are fundamentally different [35,36,60,61,62,63,64,65,66,67,68]. Ciliates are typically the dominant group in wastewater (by biomass and the number of species) while small flagellates are the most abundant form in lakewater [69]. Direct comparisons of protist diversity between human feces and both marine and freshwater aquatic environments indicated lower diversity in feces [35]. While the diversity of protists in wastewater is higher than in fecal specimens, it is still lower compared to environmental waters [47,70]. The increased diversity and richness of predatory species have been linked to elevated predator production and higher grazing rates [71] and offer a plausible explanation for our observations regarding the greater decay of FIB in the presence of protozoa indigenous to lakewater compared to those from the wastewater inoculum.

The influence of the protozoa source was not as clear for coliphages, as the predator source did not have a significant effect on the decay of F+ coliphage. Although the protozoan source significantly affected the decay of somatic coliphage, the predator source with more impact changed over time during the study, and the magnitude of difference in decay rates between predator sources was always much less than 1 log. Nonetheless, the starting concentrations of both coliphages and the decay observed in this study were comparable to an earlier field study utilizing wastewater as the source of coliphages [20], however, it was considerably lower than the studies utilizing laboratory-propagated coliphages under similar conditions [19,41]. While the preferential protozoan grazing on some viral species over others has been previously documented [38,72,73], the potential influence of coliphage source (i.e., wastewater versus laboratory cultivated strains) is novel and merits further consideration. Furthermore, while lower starting concentrations of coliphage provided a more realistic representation of levels expected in ambient waters following a wastewater pollution event, this difference could influence the observed effect (or the lack thereof) of the predatory protozoa [74,75,76]. Finally, another important distinction between our study and earlier works [20,40,41], is that we filtered lakewater and wastewater through a 0.80 µm filter, which would have retained the bacterial population from both sources, while others utilized either autoclaving or filtration through 0.22 µm, which likely resulted in their removal. The presence of the autochthonous bacterial community provided an additional abundance of potential prey for protozoan grazers, and it could have affected the time-dependent influence of protozoan source on somatic coliphage, as well as lack of predator source on F+ coliphage decay rates.

The contribution of ambient sunlight to the decay of fecal microbiota is arguably one of the best-studied environmental parameters [8,42,44]. As expected, we observed that the decay of FIB and somatic coliphages is faster under sunlight-exposed conditions compared to the shaded controls. The exception to this observation was the F+ coliphage, which decayed similarly under all treatment conditions. While F+ coliphage has been reported to be more resilient than the somatic subgroup to wastewater UV disinfection [77,78], which relies on germicidal action of UV-C spectrum [79], extrapolation to ambient sunlight conditions (mainly consisting of UV-A and UV-B spectra) [42] is not appropriate given the great differences in these two irradiation sources and intensities. Furthermore, while somatic and F+ coliphage decay rates were similar under sunlight-exposed conditions, somatic coliphage decay rates under shaded conditions were considerably lower compared to F+ (≤1.5 log_10_). This implies that consistently higher concentrations of somatic coliphage (as compared to F+) frequently detected in ambient waters [78,80,81,82,83] may be at least in part due to greater persistence of the somatic subgroup under shaded conditions (i.e., no direct exposure to ambient sunlight). This finding is novel and warrants further study, particularly as a comparison with viral pathogens.

A limited number of studies comparing decay characteristics of coliphages and viral pathogens (i.e., poliovirus-1-Sabin, norovirus GI-1, human adenovirus 2) suggest that viral indicators are more appropriate proxies for viral pathogen persistence in environmental waters than FIB [19,84,85]. We generally observed accelerated decay of both FIB compared to somatic coliphages. However, FIB decay rates under shaded conditions were similar to those of the F+ coliphages. Some viral indicators may be more resilient to environmental stressors compared to their bacterial counterparts. However, this study found that environmental conditions and the type of coliphage influence the relative rapidity of their decay rates. Previous reports also noted the extended persistence of coliphages compared to FIB in freshwaters [86,87,88] and marine environments [87,88,89]. A trend of faster decay of culturable FIB compared to viral pathogens (i.e., adenovirus 40/41 and coxsackie A9) [90,91,92,93] further supports the need for viral indicator(s).

In summary, we demonstrated that the decay of FIB in the presence of protozoa is generally more rapid than that of coliphages, further supporting the need for viral indicators of fecal pollution to better reflect the decay characteristics of pathogenic viruses in recreational waters. We have also shown that somatic coliphage decay rates under shaded conditions are considerably slower than those of F+ coliphage or FIB. This difference has important implications for the selection of coliphage groups as viral indicators for recreational water quality and highlights the need for studies that compared the decay of pathogenic viruses to that of coliphages under environmentally relevant conditions. Furthermore, we established that the lake protozoa were more influential in the decay of FIB compared to wastewater predators, though the effect of predator source on the decay of somatic coliphages, and the lack of effect of predatory source on F+ coliphage, was less clear and merits further research. While our study adds to the growing body of knowledge regarding the effect of biotic and abiotic parameters on decay, the findings should not be directly extrapolated to other geographic regions or seasons due to the inherent variability of factors such as autochthonous microbial communities, intensity, duration of UV radiation, and water temperature.

## Figures and Tables

**Table 1:** Schematic of the experimental design.

Designation	Light Intensity (Sun/Shade)	Protozoa Removed by Filtration	Overall Effects Studied
A	High (Sun)	Wastewater protozoa removed	Effect of lake protozoa on decay rates of indicators under high light intensity
B	Low (Shade)	Wastewater protozoa removed	Effect of lake protozoa on decay rates of indicators under low light intensity
C	High (Sun)	Lakewater protozoa removed	Effect of wastewater protozoa on decay rates of indicators under high light intensity
D	Low (Shade)	Lakewater protozoa removed	Effect of wastewater protozoa on decay rates of indicators under low light intensity

**Table 2:** Two-way ANOVA summary of the effect of treatment variables (predator source and sunlight) on indicator decay at each time point. Statistically significant factors are bolded.

Indicator	Time Point (days)	Factor					
		Source of Predators ^a^		Sunlight		Interaction ^b^	
		%	*p* Value	%	*p* Value	%	*p* Value
*E. coli*	T_1_	86.37	<0.0001	1.513	0.2398	4.895	0.0542
	T_3_	85.43	0.0004	0.126	0.7657	13.57	0.0224
	T_5_	81.43	0.0001	0.519	0.5561	10.65	0.0265
	T_8_	64.70	0.0008	5.350	0.1719	10.93	0.0643
	T_14_	20.45	0.0118	57.11	0.0013	18.16	0.0148
Enterococci	T_1_	96.72	<0.0001	0.033	0.7506	0.806	0.1424
	T_3_	98.46	<0.0001	0.309	0.1821	0.309	0.1821
	T_5_	75.05	<0.0001	19.53	0.0001	2.270	0.0431
	T_8_	42.59	0.0043	35.12	0.0071	0.420	0.7054
	T_14_	13.84	0.1770	59.67	0.0224	1.806	0.5950
F+ coliphage	T_1_	13.06	0.2147	13.80	0.2034	15.63	0.1787
	T_3_	1.713	0.6984	5.678	0.4854	7.708	0.4189
	T_5_	5.885	0.2175	45.59	0.0057	22.48	0.0303
	T_8_	13.81	0.1422	33.97	0.0340	10.51	0.1933
	T_14_	0.753	0.6851	19.17	0.0821	66.04	0.0100
Somatic coliphage	T_1_	54.89	0.0019	8.966	0.1028	15.00	0.0444
	T_3_	86.57	<0.0001	0.493	0.5945	0.111	0.7989
	T_5_	47.29	0.0059	25.23	0.0266	0.003	0.9775
	T_8_	10.72	0.0174	77.91	<0.0001	1.762	0.2605
	T_14_	0.775	0.6236	82.75	0.0029	0.426	0.7143

^a^ Percent contribution of each treatment variable (predator source and sunlight) to the observed variability in the dataset.^b^ Interaction between treatment variables (predator source and sunlight).

**Table 3:** Decay (cumulative log_10_ reduction ± standard deviation for independent dialysis bag replicates) values for FIB and coliphage for each treatment and sampling day.

	*E. coli*				Enterococci				F+ Coliphage				Somatic Coliphage			
	Treatment ^1^															
Days	A	B	C	D	A	B	C	D	A	B	C	D	A	B	C	D
T_1_	2.60 ± 0.19	2.39 ± 0.16	0.13 ± 0.53	0.87 ± 0.27	2.92 ± 0.10	3.10 ± 0.22	0.66 ± 0.33	0.39 ± 0.26	0.35 ± 0.11	0.34 ± 0.15	0.15 ± 0.08	0.35 ± 0.14	0.03 ± 0.04	0.02 ± 0.06	0.08 ± 0.02	0.17 ± 0.02
T_3_	2.97 ± 0.09	2.49 ± 0.07	1.10 ± 0.52	1.69 ± 0.06	3.34 ± 0.09	3.03 ± 0.18	1.19 ± 0.23	0.93 ± 0.01	1.42 ± 0.05	1.34 ± 0.14	1.40 ± 0.07	1.40 ± 0.02	0.90 ± 0.05	0.90 ± 0.05	0.70 ± 0.03	0.68 ± 0.06
T_5_	2.46 ± 0.12	2.12 ± 0.08	0.79 ± 0.47	1.33 ± 0.04	4.93 ± 0.35	3.27 ± 0.23	2.08 ± 0.21	1.27 ± 0.39	1.82 ± 0.12	1.60 ± 0.63	2.62 ± 0.21	1.34 ± 0.17	0.99 ± 0.14	1.16 ± 0.04	1.23 ± 0.15	1.40 ± 0.06
T_8_	2.99 ± 0.74	2.16 ± 0.08	1.30 ± 0.24	1.45 ± 0.11	5.86 ± 0.06	4.98 ± 0.53	4.90 ± 0.35	4.19 ± 0.43	2.48 ± 0.22	2.37 ± 0.20	2.75 ± 0.02	2.39 ± 0.10	1.57 ± 0.29	0.71 ± 0.16	2.10 ± 0.26	0.94 ± 0.10
T_14_	5.87 ± 0.18	2.70 ± 0.66	3.52 ± 0.18	2.63 ± 0.36	5.43 ± 0.12	3.44 ± 0.90	4.32 ± 1.14	2.92 ± 0.11	2.91 ± 0.06	2.85 ± 0.05	2.76 ± 0.03	2.98 ± 0.06	2.54 ± 0.06	1.27 ± 0.57	2.77 ± 0.04	1.30 ± 0.21

^1^ A (lake protozoa/sun), B (lake protozoa/shade), C (wastewater protozoa/sun), and D (wastewater protozoa/shade).

## Data Availability

Data available in a publicly accessible repository (https://catalog.data.gov/dataset/, accessed on 10 January 2023).

## Supplementary Materials

The following supporting information can be downloaded at: https://www.mdpi.com/article/10.3390/pathogens12030378/s1, Table S1. Cumulative decay rate per day values for FIB and coliphage for each treatment and sampling day. Table S2. Analyses with observations below the assay limit of detection.
