# Supplementary material for: The Effect of Protozoa Indigenous to Lakewater and Wastewater on Decay of Fecal Indicator Bacteria and Coliphage
Source: Pathogens. 2023 Feb 25;12(3):378. doi: 10.3390/pathogens12030378 (PMC10053992; doi:10.3390/pathogens12030378)
Supplement: Supplementary file 1 [file pathogens-12-00378-s001.zip › pathogens-2186311-supplementary.pdf]

Decay rate calculations

Cumulative decay rates for each indicator organism and treatment were calculated at various time points during the study as follows: $(\log_{10} C_0 - \log_{10} C_T)/T_X$ where C_T represents concentration at different sampling time points (T_1, T_3, T_5, T_8, T_{14}), C_0 represents the starting concentrations measured at T_0 and T_X represents the sampling time point in days ($T_1 = 1$; $T_3 = 3$; $T_5 = 5$; $T_8 = 8$; $T_{14} = 14$) (Table S1).

Table S1. Cumulative decay rate per day values for FIB and coliphage for each treatment and sampling day.

	E. coli				Enterococci				F+ coliphage				Somatic coliphage			
	Treatment[1]															
Days	A	B	C	D	A	B	C	D	A	B	C	D	A	B	C	D
T_1	2.60 ± 0.19	2.39 ± 0.16	0.13 ± 0.53	0.87 ± 0.27	2.92 ± 0.10	3.10 ± 0.22	0.66 ± 0.33	0.39 ± 0.26	0.35 ± 0.11	0.34 ± 0.15	0.15 ± 0.08	0.35 ± 0.14	0.03 ± 0.04	0.02 ± 0.06	0.08 ± 0.02	0.17 ± 0.02
T_3	0.99 ± 0.03	0.83 ± 0.02	0.37 ± 0.17	0.56 ± 0.02	1.11 ± 0.03	1.01 ± 0.06	0.40 ± 0.08	0.78 ± 0.82	0.47 ± 0.01	0.45 ± 0.05	0.47 ± 0.02	0.47 ± 0.01	0.30 ± 0.02	0.30 ± 0.02	0.23 ± 0.01	0.23 ± 0.02
T_5	0.49 ± 0.02	0.42 ± 0.02	0.16 ± 0.09	0.27 ± 0.01	0.85 ± 0.21	0.58 ± 0.17	0.38 ± 0.10	0.24 ± 0.11	0.36 ± 0.02	0.32 ± 0.13	0.52 ± 0.04	0.27 ± 0.03	0.20 ± 0.03	0.23 ± 0.01	0.25 ± 0.03	0.28 ± 0.01
T_8	0.37 ± 0.09	0.27 ± 0.01	0.16 ± 0.03	0.18 ± 0.01	0.73 ± 0.01	0.62 ± 0.07	0.61 ± 0.04	0.52 ± 0.05	0.31 ± 0.03	0.30 ± 0.02	0.34 ± 0.00	0.30 ± 0.01	0.20 ± 0.04	0.09 ± 0.02	0.26 ± 0.03	0.12 ± 0.01
T_{14}	0.42 ± 0.01	0.19 ± 0.05	0.25 ± 0.01	0.19 ± 0.03	0.39 ± 0.01	0.25 ± 0.06	0.31 ± 0.08	0.21 ± 0.01	0.21 ± 0.00	0.20 ± 0.00	0.20 ± 0.00	0.21 ± 0.00	0.18 ± 0.00	0.09 ± 0.04	0.20 ± 0.00	0.09 ± 0.02

[1]A (lake protozoa/sun), B (lake protozoa/shade), C (wastewater protozoa/sun), D (wastewater protozoa/shade)

Left-censored data

The frequency of left-censored observations (below assay limit of detection, also known as non-detects) was low - 1.38%-12.5% per assay out of 72 samples collected in the study (4 treatments x 3 replicates x 6 sampling time points and it mostly occurred at later time points (Table S2). The limit of detection (LOD) was substituted for left-censored observations and used to calculate $\log_{10}$ reduction and decay rate values. The LOD was 100 PFU per 100 mL for coliphages, and it varied between 1 and 10 CFU per 100 mL for enterococci, based on the volume of the sample processed. *E. coli* was always detected for all of the replicates for the duration of the study.

Table S2. Analyses with observations below assay limit of detection

Time point	Treatment	Indicator	Number of non-detects*
T_5	A	Enterococci	2/3
T_8	A		3/3
	C		2/3
T_{14}	A		1/3
	B		1/3
T_5	C	F+ coliphage	2/3
T_8	C		2/3
T_{14}	A		1/3
	C		2/3
	D		1/3
T_{14}	C	Somatic coliphage	1/3

*Out of three independent replicates.
